# HER2-Altered Non-Small Cell Lung Cancer: A Journey from Current Approaches to Emerging Strategies

**DOI:** 10.3390/cancers16112018

**Published:** 2024-05-26

**Authors:** Giorgia Ferrari, Benedetta Del Rio, Silvia Novello, Francesco Passiglia

**Affiliations:** Department of Oncology, University of Turin, San Luigi Hospital, 10124 Orbassano, Italy; giorgia.ferrari@unito.it (G.F.); benedetta.delrio@unito.it (B.D.R.); silvia.novello@unito.it (S.N.)

**Keywords:** non-small cell lung cancer, HER2, antibody-drug conjugates, targeted therapy

## Abstract

**Simple Summary:**

The introduction of trastuzumab deruxtecan is significantly changing the therapeutic landscape of advanced HER2-mutated non-small cell lung cancer (NSCLC). The results of the DESTINY-Lung04 trial are highly anticipated for their potential to redefine the first-line therapeutic standard for HER2-mutant disease. Furthermore, several studies evaluating combination therapy regimes are currently ongoing. This review outlines the current state of the art in the clinical management of HER2-altered NSCLC and explores potential future perspectives in the field of HER2 targeted strategies.

**Abstract:**

For patients diagnosed with advanced HER2-altered non-small cell lung cancer (NSCLC), the current standard of care is represented by a platinum-pemetrexed-based chemotherapy, eventually in combination with immunotherapy. Different pan-HER tyrosine kinase inhibitors have been evaluated in limited phase II trials, yielding generally unsatisfactory outcomes, although certain genotypes demonstrated some clinical benefit. Conversely, antibody-drug conjugates (ADCs) targeting HER2, particularly trastuzumab-deruxtecan, have shown promising results against HER2-mutant disease, including a great intracranial activity in patients with brain metastasis. Based on the results obtained from DESTINY-Lung01 and DESTINY-Lung02 trials, trastuzumab deruxtecan received regulatory approval as the first targeted therapy for pre-treated, HER2-mutant, advanced NSCLC patients. More recently, the Food and Drug Administration (FDA) granted the accelerated approval of trastuzumab deruxtecan for advanced, pre-treated HER2-positive solid tumours with no other treatment options. In this scenario, emerging evidence is increasingly pointing towards the exploration of combination regimens with synergistic effects in the advanced disease. In this review, we provide a detailed summary of current approaches and emerging strategies in the management of HER2-altered NSCLC, also focusing on unmet needs, including the treatment of patients with brain metastases.

## 1. Introduction

In recent years, remarkable progress has been made in non-small cell lung cancer (NSCLC) treatment by identifying oncogenic drivers and developing targeted therapies. Among these oncogenic drivers, epidermal growth factor 2 receptor (HER2) has recently emerged as a promising but challenging oncogenic driver and therapeutic target in NSCLC patients.

HER2, along with HER1 (EGFR), HER3, and HER4, is part of the HER/ERBB receptor tyrosine kinase family, which encodes receptors consisting of three domains: an extracellular domain, a transmembrane domain, and an intracellular tyrosine kinase domain. When inactive, HER receptors are in the form of monomers, and only when the ligand binding occurs is the dimerisation domain exposed, and the dimerisation allowed. The hetero- or homodimerisation between the receptors of HER family results in the activation of the downstream PI3KAKT and MEK-ERK signalling pathways to promote cell proliferation, differentiation, and migration. HER2 is the only member of the HER/erbB family that lacks a specific ligand. Consequently, it remains in a perpetually active conformational state, always making it readily available for dimerisation [1]. HER2 heterodimerises with HER1 (also known as EGFR), HER3 and HER 4 and is only able to homodimerise when overexpressed. Alterations of HER2 causing an overexpression of the receptor result in an excess of ERBB2-mediated signalling, promoting cell survival and proliferation [2,3].

HER2 alterations are present in different cancer types such as: bladder cancer, colorectal, lung, breast, and uterine cervix cancers. In recent years, anti-HER2 therapies have been developed for many of these tumours, showing great efficacy and tolerability profiles [2,4]. In this review we provide a summary of the current state of the art and future directions in the treatment of HER2-altered NSCLC.

## 2. HER2 Alterations in NSCLC

**Figure 1:**
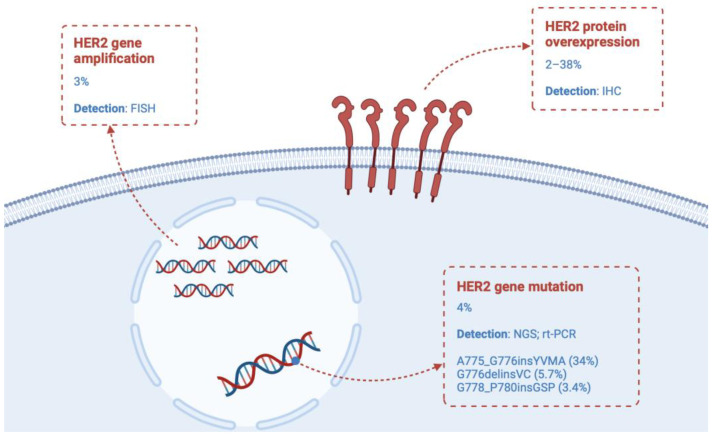
HER2 alterations in advanced non-small cell lung cancer.

Different types of HER2 alterations have been identified in NSCLC: gene mutation, gene amplification, and protein overexpression (Figure 1). The overlap between these alterations is rare. Each alteration identifies a subgroup with different biological behaviour, affecting different patient subsets and showing different responses to anti-HER2 drugs. For this reason, it is imperative to investigate them as distinct clinicopathological as well as molecular entities [5,6].

### 2.1. HER2 Mutation

HER2 mutations, occurring in approximately 4% of NSCLC and detectable by NGS or rt-PCR, are more prevalent in females, non-smokers, and adenocarcinoma histology, similar to EGFR mutations [7]. HER2 mutations are predominantly mutually exclusive with other oncogenic drivers; only a minority of patients display concurrent EGFR mutations, ALK translocations, or ROS-1 translocations [8].

The most common mutation occurs in exon 20 within the tyrosine kinase domain. It involves a 12 base-pair insertion coding for the amino acids YVMA (A775_G776insYVMA) and accounts for 34% of all HER2 mutations. Other common exon 20 mutations affecting the tyrosine kinase domain include G776delinsVC and G778_P780insGSP, which account for 5.7% and 3.4% of all HER2 mutations, respectively [9]. On the other hand, mutations such as I655V (4.5%), *P122L* (2.3%), and *G222C* (1.1%) affect the extracellular domain and S310F (5.1%) the transmembrane domain [6]. Different mutations give the disease different characteristics; the YMVA mutation is associated with a higher risk of developing brain metastases than the non-YMVA subgroup [10]. Additionally, the diverse conformational landscape of HER2 ex20ins, resulting from different mutations, contributes to the high heterogeneity in terms of clinical response to anti-HER2 agents [11].

### 2.2. HER2 Amplification

The frequency of HER2 amplification in NSCLC is approximately 3%. The most widely accepted definition of HER2 amplification is the HER2/CEP17 ratio ≥ 2.0 by fluorescence in situ hybridisation (FISH) testing, which is used in breast cancer as well as in most clinical trials. De novo amplifications of HER2 have been found to be more common in males and in smokers [12,13].

In addition, HER2 amplification has been identified as an acquired resistance mechanism in EGFR-mutant NSCLC cancers treated with EGFR inhibitors, occurring in approximately 15% of patients resistant to EGFR TKIs. HER2 amplification has been observed as a resistance mechanism to first-generation EGFR TKIs and is mutually exclusive with the more common resistance mechanism, T790M. In addition, HER2 amplification has been identified as a resistance mechanism to the third-generation EGFR inhibitor osimertinib, whether given sequentially after a first-generation inhibitor or as first-line therapy [14].

HER2-amplified disease correlates with certain particularly aggressive features, notably, larger tumour size and a higher propensity to develop pleural metastases and lymphovascular invasion. However, despite this correlation, the prognostic and predictive role of HER2 amplification in NSCLC remains unclear [15].

### 2.3. HER2 Overexpression

HER2 overexpression occurs in 2–38% of NSCLC and is generally assessed by immunohistochemistry. This alteration is predominantly observed in males and in smokers, as HER2 amplification, and in adenocarcinoma with papillary histology [12,15].

In NSCLC, HER2 overexpression is typically not driven by HER2 amplification but rather by polysomy, in contrast to breast cancer where HER2 amplification is strongly correlated with HER2 expression. Polysomy is considered to be the presence of a HER2 gene copy number greater than 5 or 6, but HER2/CEP17 < 2 [16]. However, there are conflicting findings regarding the actual overlap between overexpression and HER2 amplification [17]. While it remains unclear regarding HER2 mutations and amplifications, overexpression is associated with a worse prognosis. The reason for this correlation is not yet clear, but it may be related to increased chemoresistance resulting from this molecular alteration [13].

## 3. Current Therapeutic Approaches

### 3.1. Chemotherapy

Platinum-pemetrexed-based chemotherapy, optionally in combination with immunotherapy, currently represents the standard of care for first-line treatment of patients with HER2-altered advanced NSCLC [18]. Similar to patients harbouring KRAS and EGFR mutations, pemetrexed-based chemotherapy showed an overall response rate (ORR) of 36% and a progression-free survival (PFS) of 5.1 months [10,19].

Several clinical trials have evaluated the efficacy of chemotherapy in combination with HER2-targeted agents; these studies will be analysed in the subsequent paragraphs.

### 3.2. Immune Checkpoint Inhibitors (ICIs)

Immune checkpoint inhibitors (ICIs) have become a standard treatment option for advanced non-oncogene-addicted NSCLC. However, ICI efficacy in patients with oncogenic drivers is limited, which is attributed to a “cold” tumour microenvironment characterised by reduced PD-L1 expression and a lower tumour mutation burden (TMB) [20].

A retrospective analysis evaluating the efficacy of ICI monotherapy in patients with advanced HER2-mutant NSCLC showed an ORR of 12% with median PFS and OS of 1.9 and 10.4 months, respectively [21]. Similar outcomes were reported by the French Group on Lung Cancer (GFPC) [22] in patients who had received one previous treatment, and in the IMMUNOTARGET registry, where smoker patients with HER2-mutant NSCLC exhibited a significantly longer median PFS compared with nonsmokers (3.4 months vs. 2.0 months, *p* = 0.04) [23].

Regarding the use of ICIs in combination with chemotherapy in HER2-mutated patients in a first-line setting, the observed outcomes are similar to those of the non-selective NSCLC cohort in the KEYNOTE-189 trial with an ORR, median PFS and a one-year OS of 52%, 6 months, and 88%, respectively [24,25]. A subsequent retrospective analysis demonstrated an ORR of 28.9% and a median PFS of 5.2 months. Despite the high ORR, the increase in median PFS was not statistically significant compared to chemotherapy alone (5.2 vs. 4.03 months, *p* = 0.20) [26].

Presently, the available data do not encourage the use of ICI monotherapy for the treatment of HER2-altered advanced NSCLC. On the other hand, chemoimmunotherapy combinations, despite limited evidence, continue to be a feasible first-line treatment alternative.

### 3.3. Tyrosine Kinase Inhibitors (TKIs)

Initial efforts to target HER2 in NSCLC involved the employment of second-generation irreversible tyrosine kinase inhibitors (TKIs) designed for EGFR mutation treatment.

Afatinib was evaluated in a compassionate use program in heavily pretreated patients with HER2-mutant NSCLC and showed a median time-to-treatment failure (TTF) of 2.9 months, with interesting variations among subtypes; notably, the HER2 exon 20 YVMA insertion subtype showed a median TTF of 9.6 months [27]. Conversely, other studies identified a clinical association between HER2 exon 20 YVMA insertion and a reduced efficacy to afatinib, whereas mutations such as G778_P780dup and G776delinsVC demonstrated improved outcomes [28,29]. In the EUHER2 study, afatinib showed an ORR of 18.2% and a median PFS of 3.9 months in 11 patients with HER2-mutant NSCLC [8]. Additionally, the NICHE phase II trial confirmed the modest clinical activity of afatinib in patients with HER2-mutant NSCLC, demonstrating an ORR of 7.7% and a median PFS of 15.9 week [30].

Dacomitinib, an irreversible pan-HER TKI, was evaluated in a phase II trial in pretreated HER2-altered NSCLC patients and showed a 12% partial response rate in HER2-mutant patients, compared with 0% in those with HER2 amplification. Notably, responses were limited to patients with specific mutations (P780_Y781insGSP and M774delinsWLV) and absent in those with the A775_G776insYVMA insertion [31].

Neratinib, a pan-HER TKI, was evaluated in the phase II SUMMIT basket trial, in patients with refractory HER2-mutant NSCLC, achieving a partial response in only one patient (ORR 3.8%) [32]. In a randomised phase II trial comparing neratinib alone or in combination with temsirolimus in HER2-mutant advanced NSCLC, combination therapy achieved an ORR of 19% vs. 0% with neratinib alone [33].

Non-selective HER2-TKIs demonstrated limited efficacy in treating patients with HER2-altered NSCLC, prompting the development of next-generation TKIs to improve clinical outcomes.

Poziotinib, a novel covalent and irreversible EGFR/HER2 inhibitor, was evaluated in the ZENITH20-2 trial in NSCLC patients with HER2 exon 20 insertions. The treatment was administered at a dose of 16 mg QD and showed an ORR of 27.8% and a disease control rate (DCR) of 70.0%, achieving a median PFS of 5.5 months. However, it exhibited a suboptimal safety profile, with 97.8% of patients experiencing treatment-related adverse events (TRAEs), including 78.9% with grade 3 TRAEs [34]. With the aim of improving the drug’s tolerability profile, the ZENITH20-4 trial compared poziotinib 16 mg QD to 8 mg BID in treatment-naïve patients with HER2-mutant NSCLC. The study revealed an ORR of 41% and a median PFS of 5.6 months, and it found that the twice-daily regimen led to fewer dose reductions and interruptions compared to the once-daily regimen, while maintaining a comparable incidence of grade ≥ 3 TRAEs [35]. In consideration of the complex evaluation of the overall risk-benefit analysis, the FDA has determined that additional data from a randomised controlled trial are needed before poziotinib can be approved in pre-treated patients with NSCLC harboring HER2 exon 20 insertion mutations.

Pyrotinib, an irreversible pan-HER TKI, was evaluated in a phase II trial including platinum-pretreated advanced NSCLC patients, achieving an ORR of 30% and a median PFS of 6.9 months. Subgroup analyses revealed that patients with different HER2 mutations, including those with BMs, benefited from the treatment [36]. In a different phase II trial, pyrotinib was administered as either first or subsequent line in patients with advanced HER2-mutant NSCLC, yielding an ORR of 19.2% and a median PFS of 5.6 months. Notably, treatment-naive patients experienced a higher median PFS (8.9 months) [37]. Additionally, the PATHER2 phase II trial evaluated pyrotinib in combination with apatinib in pretreated advanced HER2-altered NSCLC patients, showing an ORR of 51.5%, a median PFS of 6.9 months, and a median OS of 14.8 months [38]. New efficacy and safety data will be provided by the ongoing phase III PYRAMID-1 trial comparing pyrotinib with docetaxel in patients with advanced NSCLC harbouring a HER2 exon 20 mutation who have been previously treated with platinum-based chemotherapy (NCT04447118).

Mobocertinib (TAK-788), an oral EGFR/HER2 inhibitor targeting exon 20 insertions, demonstrated antitumor activity in a phase I/II trial involving advanced NSCLC patients with EGFR exon 20 insertion, while results from an expansion cohort focusing on patients harbouring HER2 exon 20 alterations are still pending [39,40].

Currently, phase I and II clinical studies are evaluating other non-selective HER2 TKIs, including furmonertinib (NCT05364073), BAY2927088 (NCT05099172), and tucatinib in combination with trastuzumab (NCT04579380).

Ongoing efforts are also focused on developing novel and highly-HER2 selective TKIs, with the aim of improving treatment efficacy. BI 1810361 (zongertinib), a potent covalent HER2 inhibitor, demonstrated an ORR of 46% in an ongoing phase I trial (NCT04886804) involving HER2-mutant NSCLC patients refractory to platinum-based chemotherapy [41]. Furthermore, ELVN-002 has demonstrated efficacy against common HER2 mutations along with the ability to penetrate the CNS [42] and is currently under evaluation in a phase I clinical trial (NCT05650879). Table 1 lists the major ongoing clinical trials of TKIs in HER2-altered NSCLC.

### 3.4. Monoclonal Antibodies

Trastuzumab, a humanised IgG monoclonal antibody targeting HER2 directly, has been evaluated for its efficacy in advanced NSCLC patients with HER-2/neu positivity across various chemotherapy regimens. Its addition to carboplatin and paclitaxel in a Phase II clinical trial demonstrated an ORR of 24.5%, with a median PFS and OS of 3.3 and 10.1 months, respectively [43]. Subgroup analysis revealed improved survival outcomes in patients with HER-2/neu 3+ expression, consistent with findings from other chemotherapy regimens such as cisplatin-gemcitabine [44]. However, the limited number of HER2 3+ or FISH-positive patients precludes a definitive confirmation of these results.

Trastuzumab monotherapy was evaluated in the phase II HOT1303-B trial, which enrolled pre-treated HER2-altered NSCLC patients and resulted in no therapeutic response (ORR 0%), although it achieved a disease control ratio (DCR) of 70.0% and a median PFS of 5.2 months [45].

The combination of trastuzumab and pertuzumab showed a modest ORR of 11% in heavily pre-treated patients with HER2-mutant or amplified NSCLC, particularly in those harbouring HER2 exon 20 mutations [46].

Given the favourable results observed with the combination of trastuzumab, pertuzumab, and docetaxel in breast cancer, its efficacy was assessed in the single-arm phase II IFCT 1703-R2D2 trial in patients with HER2-mutant NSCLC who had previously been treated with platinum-based chemotherapy. This regimen demonstrated an ORR of 29% and a median PFS of 6.8 months, with equally promising results [47]. Notably, this combination demonstrated a median duration of response (mDoR) of 11.0 months, significantly exceeding the duration of response observed with other HER2-targeted monoclonal antibody regimens.

### 3.5. Antibody-Drug Conjugates (ADCs)

Trastuzumab Emtansine (T-DM1) is an antibody-drug conjugate (ADC) combining the HER2-targeted monoclonal antibody trastuzumab with the cytotoxic microtubule inhibitor emtansine (DM1). In a phase II basket trial, T-DM1 was administered at a dose of 3.6 mg/kg to 18 heavily pre-treated HER2-mutant patients with advanced NSCLC, achieving an ORR of 44% and a PFS of 5 months. Biomarker analysis identified variations in response rates by HER2 mutation subtype, with the highest observed in exon 20 insertion mutations, and no significant correlation between HER2 IHC and clinical outcomes [48]. Updated analyses involving 28 HER2-mutant and 11 HER2-amplified pre-treated NSCLC patients revealed an ORR of 50% and 55%, respectively [49]. A study conducted on patients with HER2-overexpressed NSCLC treated with T-DM1 reported no therapeutic response in the IHC 2+ group and an ORR of 20% in the IHC 3+ group, despite a similar PFS and OS. Further analysis indicated that most responders had HER2 amplification, with a subset also showing HER2 mutations, highlighting the insufficiency of exclusively relying on IHC to forecast the efficacy of T-DM1, differently from what is observed in breast and gastric cancer [50]. In conclusion, a cohort of 22 NSCLC patients with HER2 exon 20 insertion mutations was evaluated in a phase II study revealing an ORR of 38.1% and a median PFS of 2.8 months, highlighting the limited response duration associated with T-DM1 treatment [51].

Trastuzumab deruxtecan (T-DXd) is a novel anti-HER2 antibody-drug conjugate (ADC) consisting of deruxtecan, a topoisomerase I inhibitor, conjugated to trastuzumab via a cleavable linker. In the phase III DESTINY-Breast03 trial, T-DXd achieved an ORR of 79% compared to 35% with standard treatment in patients with unresectable or metastatic HER2-positive breast cancer previously treated with anti-HER2 regimens, leading to its FDA and EMA approval [52]. Based on these encouraging results, its efficacy has also been studied in patients with HER2-altered NSCLC. In a phase I study, T-DXd showed promising activity in patients with HER-altered NSCLC, with an ORR of 55.8% and a median PFS of 11.3 months. Of particular note was the improved efficacy seen in the HER2-mutant subgroup, with an ORR of 72.7% [53]. Thereafter, the phase II DESTINY-Lung01 trial evaluated the efficacy and safety of T-DXd at the dose of 6.4 mg/kg in HER2-mutant or overexpressed, recurrent, or refractory NSCLC. Notable outcomes were seen in the HER2-mutant subgroup, where T-DXd monotherapy showed an ORR of 55%, median PFS of 8.2 months, and median OS of 17.8 months [54]. These findings, supported by preclinical research showing enhanced receptor internalisation and trastuzumab deruxtecan intracellular uptake in activating HER2 mutations, confirm the heightened activity of trastuzumab deruxtecan in HER2-mutant versus HER2-overexpressing NSCLC [49]. The toxicity profile of T-DXd was characterised by a 97% incidence of TRAEs, 46% of which were grade ≥ 3, leading to dose interruption, reduction, or treatment discontinuation in 53.1%, 34.7%, and 22.4% of cases, respectively. Notably, interstitial lung disease (ILD) emerged as the main adverse effect, affecting 26% of the patient population and leading to two deaths [54]. The greater frequency of ILD observed in lung cancer versus breast or gastric cancers may reflect underlying pulmonary conditions in lung cancer patients, including smoking-related damage or reduced lung capacity from prior treatments, but further investigation for definitive conclusions is needed [55]. The subsequent phase II DESTINY-Lung02 trial revealed that for HER2-mutant, pre-treated NSCLC patients, a 5.4 mg/kg Q3W dosage of T-DXd outperforms the 6.4 mg/kg Q3W regimen, achieving higher ORR (53.8% vs. 42.9%), with reduced severe TRAEs (31.7% vs. 58%) and ILD incidence (5.9% vs. 14%) [56]. These results led to the FDA and EMA approval of trastuzumab deruxtecan as the first and only approved targeted therapy for the treatment of metastatic NSCLC with HER2 mutations previously treated with platinum-based chemotherapy. Moreover, on 5 April 2024, the FDA granted accelerated approval to T-DXd for patients with advanced, pre-treated HER2-positive (IHC3+) solid tumours with no other treatment options.

T-DXd is currently under evaluation also in first-line setting. The ongoing phase III DESTINY-Lung04 trial (NCT05048797) will assess the efficacy and safety of T-DXd as single-agent therapy compared to chemotherapy plus pembrolizumab for first-line treatment in advanced NSCLC patients with HER2 exon 19 or 20 mutations.

## 4. Brain Metastases in HER2-Altered NSCLC: Incidence and Treatment Strategies

The occurrence of brain metastases in patients with HER2-altered NSCLC ranges from 6% to 29% [8,57,58], with exon 20 YVMA insertion associated with a significantly higher baseline and lifetime incidence [10]. It is, therefore, undeniable that the development of HER2-targeted therapies capable of crossing the blood-brain barrier (BBB) is necessary to achieve more effective treatments and longer lasting responses over time.

As previously mentioned, although non-selective HER2 TKIs like afatinib, dacomitinib, and pyrotinib showed systemic efficacy in HER2-mutant NSCLC, data on their impact on the central nervous system (CNS) are lacking. On the other hand, poziotinib revealed an ORR of 28.6% and a median PFS of 7.4 months in patients with BMs, but the reliability of these results is limited by a small cohort size, the omission of a baseline brain MRI, and the prevalent use of prior brain radiation [59].

T-DXd demonstrated significant intracranial efficacy in HER2-positive breast cancer. In the phase III DESTINY-Breast03 trial, T-DXd achieved an intracranial response rate of 63.8%, compared to 33.3% for T-DM1 among patients with stable BMs at baseline [60]. Furthermore, a subgroup analysis from DESTINY-Breast01 recently underscored the efficacy of T-DXd on stable BMs in patients previously treated with T-DM1 [61]. T-DXd demonstrated promising efficacy also in the treatment of HER2-positive metastatic breast cancer patients with active BMs. In this scenario, the phase II DEBBRAH trial recorded an intracranial response rate of 44.4% among individuals treated with T-DXd with either HER2-positive or HER2-low breast cancer experiencing progression of BMs after local therapy [62]. Moreover, in the phase II TUXEDO-1 trial, T-DXd achieved an intracranial response rate of 100% in patients with de novo and 66.7% in patients with progressive BMs [63].

Consistent with what observed in breast cancer, T-DXd exhibited encouraging intracranial activity also in patients with HER2-mutant NSCLC. Specifically, a pooled analysis of the DESTINY-Lung01 and DESTINY-Lung02 trials demonstrated that 86% of patients with measurable BMs receiving a 5.4 mg/kg dose and 78% receiving a 6.4 mg/kg dose of T-DXd showed a reduction in brain lesions size [54,56]. Moreover, the ongoing DESTINY-Lung04 trial (NCT05048797) will evaluate CNS PFS as secondary endpoint.

## 5. Future Directions in HER2-Positive NSCLC Treatment

The treatment of HER2-driven NSCLC is expected to evolve from monotherapy to combinations of agents with synergistic effects, significantly changing the current therapeutic landscape.

Preclinical studies have revealed that T-DXd increases the expression of PD-L1 by the major histocompatibility complex class I and potentiates the infiltration of CD8 T cells into breast cancer cells, outlining the therapeutic potential of the combination of T-DXd and ICI [64]. In light of these findings, several clinical trials are underway. The phase II HUDSON basket trial evaluated the combination of T-DXd plus durvalumab in patients previously treated with anti-PD1/PD-L1 therapy, including those with HER2-mutant NSCLC and HER2-overexpressed NSCLC, showing greater efficacy in the former subgroup [65]. The phase Ib DESTINY-Lung03 trial (NCT04686305) is evaluating the safety, tolerability and efficacy of T-DXd in combination with durvalumab and chemotherapy as first-line treatment, but only in patients with advanced HER2-overexpressed NSCLC [66]. In addition, an ongoing phase Ib trial (NCT04042701) is investigating T-DXd in association with pembrolizumab in HER2-positive and mutant NSCLC patients without previous exposure to anti-PD-1/PD-L1 or HER2 therapy. However, the concurrent administration of Durvalumab and T-DXd, both associated with pulmonary toxicity, presents significant risks. In the HUDSON trial, 55% of patients experienced grade ≥ 3 TEAEs, including pneumonitis, pulmonary embolism, and anaemia, with pneumonitis being the most prevalent [65].

ICIs are also under evaluation in combination with TKIs in HER2-mutant NSCLC after failure of first-line chemotherapy, in a phase II study involving pyrotinib and PD-1 inhibitors (NCT04144569). The combination of HER2 TKIs and ICIs is supported by their synergistic effects: TKIs induce immunogenic cell death and cytokine release, enhancing tumour antigen presentation and immune cell activation; on the other hand, ICIs amplify the immune response by preventing T-cell inactivation, resulting in a more effective antitumour response [67].

Improving the architectural configuration of ADCs to amplify their efficacy and reduce associated toxicities is a promising direction. Within this framework, novel HER2-targeted ADCs such as A166, ARX788, SHRA1811, and MRG002 are currently under evaluation in phase I/II trials in patients with HER2-altered NSCLC (NCT03602079, NCT03255070, NCT04818333, and NCT05141786). Additionally, an ongoing phase IB/II clinical trial will evaluate SHR-A1811 in combination with pyrotinib or SHR-1316 in patients with HER-2 altered advanced NSCLC (NCT05482568). In conclusion, the NCT04235101 trial is exploring the safety profile of the combination therapy involving SYD985 (trastuzumab duocarmazine) and niraparib in individuals diagnosed with solid tumours. Table 2 lists the main ongoing clinical trials of ADCs in HER2-altered NSCLC.

Emerging evidence highlights the importance of liquid biopsy in monitoring treatment efficacy, with recent studies showing that fluctuations in circulating tumour DNA (ctDNA) levels correlate with treatment outcomes and survival rates in solid tumours undergoing targeted therapy [68].

Consistent with previous findings, a recent analysis of advanced HER2-mutant NSCLC patients receiving pyrotinib from two phase II clinical trials reported improved treatment outcomes in those with ctDNA clearance after 40 days of therapy. However, the limited number of patients enrolled in the analysis makes further investigation necessary [69]. Finally, an interesting case report showed how the kinetics of HER2 mutation allele frequency assessed by plasma ctDNA analysis at different timepoints during treatment with T-DXd exactly matched the clinical course of the disease [70].

## 6. Conclusions

This review highlights the dynamic and evolving landscape of HER2-targeted therapies in NSCLC, marking significant advances from the initial use of monotherapies to more recent strategies of combination regimen. While platinum-pemetrexed-based chemotherapy still represents the standard of care for first-line treatment, emerging therapies, including T-DXd and novel TKIs, showed promising results also in treating patients with BMs. Notably, the outcomes of the DESTINY-Lung04 trial are keenly awaited as they hold the potential to significantly alter the treatment paradigm for patients with advanced HER2-mutant NSCLC. Moreover, emerging evidence is pointing to the potential role of liquid biopsy in monitoring disease progression, but further studies are needed.

## Figures and Tables

**Table 1:** Ongoing clinical trial of TKIs in HER2-altered NSCLC.

Trial (ClinicalTrials.gov Identifier)	Description	Phase	Drug	HER2 Alteration
NCT05378763	A Study of Poziotinib in Previously Treated Participants With Locally Advanced or Metastatic NSCLC Harboring HER2 Exon 20 Mutations (PINNACLE)	III	Poziotinib 8 mg BID; Docetaxel 75 mg/mq	Exon 20 mutations
NCT04447118	Phase 3 Study of Pyrotinib Versus Docetaxel in Patients With Advanced Non-squamous NSCLC Harboring a HER2 Exon 20 Mutation Who Failed Platinum Based Chemotherapy (PYRAMID-1)	III	Pyrotinib 400 mg QD; Docetaxel 75 mg/mq	Exon 20 mutations
NCT05364073	Study of Furmonertinib in Patients With Advanced or Metastatic Non-Small Cell Lung Cancer (NSCLC) With Activating, Including Uncommon, Epidermal Growth Factor Receptor (EGFR) or Human Epidermal Growth Factor Receptor 2 (HER2) Mutations	Ib	Furmonertinib	Exon 20 mutation; EGFR exon 20 mutations and uncommon mutations
NCT05099172	First in Human Study of BAY2927088 in Participants Who Have Advanced Non-small Cell Lung Cancer (NSCLC) With Mutations in the Genes of Epidermal Growth Factor Receptor (EGFR) and/or Human Epidermal Growth Factor Receptor 2 (HER2)	I	BAY2927088	HER2 mutations; EGFR mutations
NCT04579380	Basket Study of Tucatinib and Trastuzumab in Solid Tumors With HER2 Alterations	II	Tucatinib 300 mg BID + Trastuzumab 6 mg/kg	HER2 mutations, overexpression, or amplification
NCT04886804	Beamion LUNG-1: An Open Label, Phase I Dose Escalation Trial, With Dose Confirmation and Expansion, of Zongertinib (BI 1810631) as Monotherapy in Patients With Advanced or Metastatic Solid Tumors With HER2	I	Zongertinib	HER2 mutations, overexpression, or amplification
NCT05650879	A Phase 1a/1b Study of ELVN-002 for the Treatment of Patients With HER2 Mutant Non-Small Cell Lung Cancer	I	ELVN-002	HER2 mutations
NCT04144569	PD-1 Combined With Pyrotinib for Chemotherapy Failure HER2 Insertion Mutation Advanced NSCLC	II	PD-1 + Pyrotinib 400 mg QD	HER2 insertion mutations

Abbreviations: TKI, tyrosine kinase inhibitors; NSCLC, non-small cell lung cancer; QD, once daily; BID, twice daily.

**Table 2:** Ongoing clinical trial of ADCs in HER2-altered NSCLC.

Trial (ClinicalTrials.gov Identifier)	Description	Phase	Drug	HER2 Alteration
NCT05048797	An Open-label, Randomized, Multicenter, Phase 3 Study to Assess the Efficacy and Safety of Trastuzumab Deruxtecan as First-line Treatment of Unresectable, Locally Advanced, or Metastatic NSCLC Harboring HER2 Exon 19 or 20 Mutations (DESTINY-Lung04)	III	T-DXd	Exon 19 or 20 mutations
NCT04686305	A Phase Ib Multicenter, Open-label Study to Evaluate the Safety and Tolerability of Trastuzumab Deruxtecan (T-DXd) and Immunotherapy Agents With and Without Chemotherapy Agents in First-line Treatment of Patients With Advanced or Metastatic Non-squamous Non-small Cell Lung Cancer (NSCLC) and Human Epidermal Growth Factor Receptor 2 (HER2) Overexpression (OE) (DESTINY-Lung03)	Ib	T-DXd; T-DXd + Durvalumab + Cisplatin, Carboplatin or Pemetrexed; T-DXd + MEDI5752; T-DXd + MEDI5752 + Carboplatin	HER2 overexpression
NCT04042701	A Phase 1b, Multicenter, Two-Part, Open-Label Study of Trastuzumab Deruxtecan (DS-8201a), An Anti-Human Epidermal Growth Factor Receptor-2 (HER2)-Antibody Drug Conjugate (ADC), In Combination With Pembrolizumab, An Anti-PD-1 Antibody, For Subjects With Locally Advanced/Metastatic Breast Or Non-Small Cell Lung Cancer (NSCLC)	Ib	T-DXd + Pembrolizumab	HER2-expressing, HER2 mutations
NCT03602079	A Phase I-II, FIH Study of A166 in Locally Advanced/Metastatic Solid Tumors Expressing Human Epidermal Growth Factor Receptor 2 (HER2) or Are HER2 Amplified That Did Not Respond or Stopped Responding to Approved Therapies	I-II	A166	HER2-expressing, HER2 amplification
NCT03255070	A Phase 1, Multicenter, Open-label, Multiple Dose-escalation and Expansion Study of ARX788, as Monotherapy in Advanced Solid Tumors With HER2 Expression	I	ARX788	HER2-expressing
NCT04818333	Phase I/II Clinical Study of the Safety, Tolerability, Pharmacokinetics, and Efficacy of SHR-A1811 for Injection in Subjects With Advanced Non-small Cell Lung Cancer Who Have HER2 Expression, Amplification, or Mutation	I	SHR-A1811	HER2-expresssing, HER2 amplification, HER2 mutations
NCT05141786	An Open-label, Multi-center, Non-randomized Phase II Clinical Study to Evaluate the Efficacy and Safety of MRG002 in Patients With HER2-mutated Unresectable/ Metastatic Non-small Cell Lung Cancer (NSCLC)	II	MRG002	HER2 mutations
NCT05482568	Phase IB/II Clinical Study of the Safety, Tolerability, Pharmacokinetics, and Efficacy of Injectable SHR-A1811 in Combination With Pyrotinib or SHR-1316 in Subjects With Advanced Non-small Cell Lung Cancer With HER2	Ib/II	SHR-A1811 + Pyrotinib; SHR-A1811 + SHR-1316	HER2-expresssing, HER2 amplification, HER2 mutations
NCT04235101	A Two-part Phase I Study With the Antibody-drug Conjugate SYD985 in Combination With Niraparib to Evaluate Safety, Pharmacokinetics, and Efficacy in Patients With HER2-expressing Locally Advanced or Metastatic Solid Tumours	I	SYD985 + Niraparib	HER2-expressing

Abbreviations: ADC, antibody; NSCLC, non-small cell lung cancer; T-DXd, trastuzumab deruxtecan.
